# Paediatric Major Trauma: A Retrospective Observational Comparison of Mortality in Prehospital Bypass and Secondary Transfer in the East of England

**DOI:** 10.7759/cureus.36808

**Published:** 2023-03-28

**Authors:** Owen Hibberd, James Price, Amy Laurent, Shruti Agrawal, Ed Barnard

**Affiliations:** 1 Department of Emergency Medicine, Blizard Institute, Queen Mary University of London, London, GBR; 2 Department of Emergency, Addenbrooke's Hospital, Cambridge University Hospitals NHS Foundation Trust, Cambridge, GBR; 3 Department of Research, Audit, Innovation, and Development (RAID), East Anglian Air Ambulance, Norwich, GBR; 4 Department of Paediatric Intensive Care, Addenbrooke's Hospital, Cambridge University Hospitals NHS Foundation Trust, Cambridge, GBR; 5 Department of Paediatrics, University of Cambridge, Cambridge, GBR; 6 Academic Department of Military Emergency Medicine, Royal Centre for Defence Medicine (Research & Clinical Innovation), Birmingham, GBR

**Keywords:** trauma, patient transfer, retrieval, pre-hospital, paediatric emergency medicine

## Abstract

Background

More than half of seriously injured children are not initially treated at a major trauma centre (MTC). Children may be transported by private vehicle to a trauma unit (TU). Children may also be transported by emergency medical services (EMS) to the nearest TU with approximately one in five of these undergoing secondary transfer to an MTC. Most trauma networks permit TU bypass to an MTC. However, the evidence on outcomes between transfer and bypass is limited.

This study aimed to evaluate the use of the trauma network by comparing outcomes between paediatric major trauma patients by the method of presentation.

Methods

In this retrospective observational study, a consecutive sample of paediatric (<16 years old) major trauma patients transported to the regional MTC (Cambridge University Hospitals NHS Foundation Trust (CUH)) between 1st January 2015 and 31st December 2020 was included. Patients were excluded if they arrived at the MTC >24 hours post-injury or were transported to the MTC as the nearest hospital. Patients were divided into four groups: self-presented to MTC, MTC as nearest hospital, bypass and secondary transfer.

Results

A total of 315 patients (28 ‘self-presented’, 55 ‘nearest’, 58 ‘bypass’ and 174 ‘secondary transfers’) were included. The median age was 9.4 [3.7-13.6] years, and *n*=209 (66.3%) were male. The median Injury Severity Score (ISS) was 16.0 [9.0-25.0] and *n*=190 (60.3%) had an ISS >15.

There was no difference in 30-day mortality between the ‘bypass’ and ‘secondary transfer’ groups. There was a significantly longer hospital and intensive care unit length of stay (LOS) in the bypass group compared to other groups, both *p*<0.001. The median time to definitive care was five hours greater in the secondary transfer group compared to ‘bypass’ (bypass 117.6 minutes [100.8-136.6], secondary transfer 418.8 minutes [315.6-529.8]).

Conclusion

There was no significant difference in 30-day mortality of paediatric major trauma patients who underwent secondary transfer compared to those transported directly from the scene to the MTC, despite significant time delays in reaching definitive care.

## Introduction

In the United Kingdom (UK), trauma is a leading cause of morbidity and mortality in children [1]. Since 2012, and the introduction of trauma networks in England, there has been a significant reduction in the overall mortality of seriously injured trauma patients [2]. However, less than half of all major trauma patients are transported directly from the scene of an incident to a major trauma centre (MTC), with one in five patients undergoing secondary transfer from a trauma unit (TU) to an MTC [3]. This effect is more pronounced in the paediatric population. More than half of seriously injured children are not initially treated at an MTC. Children may be transported by caregivers in a private vehicle. Children may also be transported by emergency medical services (EMS) to the nearest hospital, which by geography is often a TU, and later require a secondary transfer to a centre capable of providing definitive care [4].

To ensure that children are not disadvantaged by the phenomenon of ‘presenting to the wrong hospital’, specialist paediatric transfer teams have been established to assist in the stabilisation and transfer of patients to an MTC [5]. Adult patients who undergo secondary transfer are more likely to have a delay in time to imaging and surgery, and have an increased mortality compared to those transported to an MTC directly, which may be confounded by utilising the TU as a stabilising centre before transfer to the MTC [2,3]. However, outcome data on the impact of secondary transfer compared to direct transported to an MTC in paediatric major trauma are limited. Standards from the UK Paediatric Intensive Care Society (PICS) specify that paediatric transfer teams should reach the bedside within three hours of referral [6,7]. Recent UK studies report an increase in intensive care unit (ICU) length of stay (LOS) with increasing ‘time-to-bedside’ of the paediatric transfer team for secondary transfers, but no evidence of effect on 30-day mortality [7-9].

These studies do not report outcomes against time interval since injury or focus on time-critical paediatric trauma as they utilise time from the transfer team request [8,9]. Therefore, the impact of EMS TU-bypass decisions on outcomes in paediatric trauma is unknown. This evaluation of the trauma network hypothesizes that paediatric patients who are transferred will have a higher mortality, increased hospital and ICU LOS, compared to those transported directly from the scene to an MTC (bypassing a local TU). This study aims to describe the paediatric trauma population in a large geographic region of the UK.

## Materials and methods

The East of England trauma network

The East of England is one of the largest geographical regions in England, operating an inclusive trauma network, serving a population of over six million people [10]. The network comprises 12 TUs feeding into a single combined adult/paediatric MTC in Cambridge. The East of England Ambulance Service NHS Trust (EEAST) is the EMS for the region [11]. EMS personnel use a regional major trauma triage tool (MTTT) to determine the most appropriate destination for trauma patients. This tool directs EMS to transport those triggering the MTTT (most seriously injured patients) directly to the MTC if the estimated transfer time is ≤45 minutes, bypassing the nearest TU [10]. For patients with critical needs, despite triggering the MTTT, EMS are instructed to transport patients to the nearest TU for stabilization [11]. In addition, permission to bypass in other situations can be requested through communication with the Network Co-ordination Service (NCS) which is led by an emergency medicine consultant; in practice, this method is only regularly utilized by the regional physician-led Helicopter Emergency Medical Services (HEMS) [12].

Study population

The national Trauma Audit Research Network (TARN) database includes patients of all ages who sustain an injury requiring admission for at least three days, admission to ICU, transfer to an MTC, or death within 30 days [5]. Individual injuries are assigned an Abbreviated Injury Scale (AIS) score, ranging from 1 (minor injury) to 6 (an injury that is thought to be ‘incompatible with life’) [5]. The Injury Severity Score (ISS), derived by adding the squares of the three highest-scoring body regions, ranges from 1 to 75, with major trauma defined as ISS >15 [13]. Data are entered into the TARN database by trained local coordinators using retrospective chart review methodology. Case ascertainment for the study period was 100%.

Inclusion criteria

A retrospective cohort study was undertaken. A consecutive sample of paediatric (aged <16 years) TARN-positive major trauma patients transported to the regional MTC (Cambridge University Hospitals NHS Foundation Trust (CUH)) by EMS between 1st January 2015 and 31st December 2020 were included [14].

Exclusion criteria

Patients were excluded if they arrived at the MTC >24 hours post-injury. Patients with incomplete data and those who were discharged without medical consent were also excluded.

Definitions

Patients were divided into four groups based on their pathway from the time of incident to definitive care: self-presented to the MTC (‘self-presented’ group), EMS transport to the MTC as the nearest hospital (‘nearest’ group), EMS transfer to the MTC bypassing the nearest TU (‘bypass’ group), and those who were initially transported from scene to a TU and then underwent secondary transfer to the MTC (‘secondary transfer’ group).

Data collection

Demographic, mechanism of injury, time of injury, anatomical injury severity (AIS), injury severity score (ISS), time to hospital (injury to hospital arrival interval), time to definitive care (injury to MTC arrival interval), HEMS attendance at the scene, hospital LOS, ICU LOS, 30-day mortality, and functional outcome (Glasgow Outcome Scale (GOS)) were extracted from the CUH TARN Trauma Office records. Two independent authors used a standardised template (available as supplementary material) to review the data and reduce misclassification bias. Patient electronic medical records (Epic Hyperspace Production®, Epic Systems Corporation, Verona, WI, USA) were interrogated to capture EMS records and scanned patient records from the TU (if applicable).

Primary outcome

The primary outcome was a comparison of 30-day mortality, in keeping with previous research.

Secondary outcomes

The secondary outcomes were to compare the functional neurological outcome (GOS), hospital LOS, ICU LOS, and time interval to definitive care.

Statistical analysis

Basic demographic, mechanism of injury, and injury data have been reported as number (percentage) and median [interquartile range, IQR]. To compare two proportions this study used Fisher’s exact test reported with a Baptista-Pike calculated odds ratio (OR) with 95% confidence interval (95% CI - Wilson/Brown method), and a p-value. Normally-distributed data have been compared with an unpaired, two-tailed Student’s t-test (with Welch’s correction for unequal standard deviations), and reported as a p-value. Non-normally distributed data have been compared with a Mann-Whitney U test and reported as a p-value. Data were analysed in Prism for macOS (v.9.4.1, GraphPad Software, San Diego, CA, USA).

Ethical review

The study met National Institute for Health Research (NIHR) criteria for service evaluation and was registered with the Cambridge University Hospitals NHS Foundation Trust Audit, Quality and Safety Department (ID3298). Strengthening The Reporting of Observational Studies in Epidemiology (STROBE) reporting guidelines were followed [15]. There was no patient or public involvement in the design or delivery of the study.

## Results

**Figure 1:**
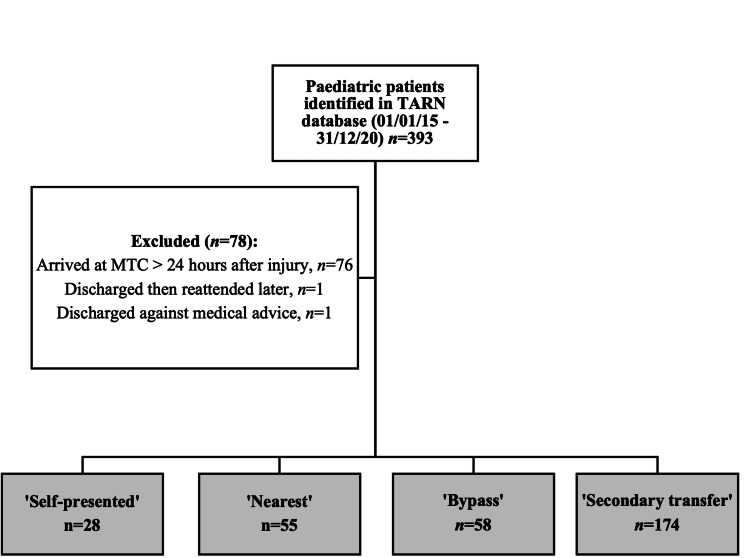
Paediatric trauma patients managed in the East of England Major Trauma Centre (MTC), 2015-2020, by mode of presentation TARN: Trauma Audit Research Network, MTC: Major Trauma Centre, TU: Trauma Unit, EMS: Emergency Medical Services. ‘Self-presented’ – transported by private vehicle to MTC ‘Nearest’ – EMS transport to MTC as nearest hospital ‘Bypass’ – primary EMS transported to the MTC, bypassing the nearest TU. ‘Secondary transfer’ – initially transported from the scene to a TU and then underwent secondary transfer to the MTC.

During the study period, n=393 paediatric trauma patients were identified. Following 78 protocol exclusions, 315 patients were included in the final analysis: 28 ‘self-presented’, 55 ‘nearest’, 58 ‘bypass’ and 174 ‘secondary transfers’ (Figure 1).

Overall study population

The median age was 9.4 [3.7-13.6] years, and n=209 (66.3%) were male. The median ISS was 16.0 [9.0-25.0] and n=190 (60.3%) had an ISS >15. Overall the most prevalent mechanism of injury was motor vehicle collision (MVC), n=141 (44.8%). Serious or more severe anatomical injury (AIS ≥3) was most often present in the head, followed by limbs, abdomen, and chest (Table 1).

**Table 1:** Patient demographics and injury details by mode of presentation for paediatric trauma patients managed in the East of England major trauma centre (2015-2020), n=315 IQR: Interquartile Range, ISS: Injury Severity Score, AIS: Abbreviated Injury Scale. * = p<0.05, ** = p<0.01

	Self-presented	Nearest	Bypass	Secondary transfer
n (% of group)	28 (8.9%)	55 (17.5%)	58 (25.0%)	174 (75.0%)
Age (years) / median [IQR]	7.4 [1.8-10.3]	9.8 [3.7-13.8]	9.4 [5.3-13.5]	10.0 [3.8-13.7]
Male sex / n (%)	15 (53.6%)	38 (69.1%)	37 (63.8%)	119 (68.4%)
ISS / median [IQR]	9.0 [9.0-10.8]	9.0 [9.0-18.0]	20.0 [10.8-29.0]	16.0 [10.0-25.0]
ISS >15 / n (%)	7 (25.0%)	21 (38.2%)	40 (69.0%)	122 (70.1%)
Mechanism of injury / n (%)				
Motor vehicle collision	8 (28.6%)	21 (38.2%)	41 (70.7%)	71 (40.8%)
Fall <2m	18 (64.3%)	18 (32.7%)	3 (5.2%)	48 (27.6%)
Fall >2m	2 (7.1%)	3 (5.5%)	5 (8.6%)	21 (12.1%)
Blows	0	6 (10.9%)	1 (1.7%)	19 (10.9%)
Penetrating	0	2 (3.6%)	3 (5.2%)	4 (2.3%)
Other (incl. burn)	0	0	5 (8.6%)	11 (6.3%)
AIS ≥3 injury / n (%)				
Head	4 (14.3%)	16 (29.1%)	31 (53.4%)	96 (55.2%)
Chest	0	8 (14.5%)	21 (36.2%)	27 (15.5%)
Abdomen	8 (28.6%)	6 (10.9%)	7 (12.1%)	38 (21.8%)
Pelvis	0	2 (3.6%)	4 (6.9%)	6 (3.4%)
Limbs	11 (39.3%)	25 (45.5%)	12 (20.7%)	15 (8.6%)
Spine	0	2 (3.6%)	1 (1.7%)	9 (5.2%)
Other (incl. face)	0	6 (10.9%)	8 (13.8%)	6 (3.5%)
Head AIS / n (%)				
0	23 (82.1%)	38 (69.1%)	24 (41.4%)	73 (42.0%)
1	0	0	2 (3.4%)	2 (1.1%)
2	1 (3.6%)	1 (1.8%)	1 (1.7%)	3 (1.7%)
3	2 (7.1%)	3 (5.5%)	7 (12.1%)	16 (9.2%)
4	1 (3.6%)	7 (12.7%)	13 (22.4%)	40 (23.0%)
5	1 (3.6%)	6 (10.9%)	11 (19.0%)	40 (23.0%)
Mean number of AIS ≥3 body region injuries / n (±sd)	0.8 (±0.4)	1.2 (±.0.6)	1.5 (±0.9)	1.1 (±0.6)

The most prevalent mechanism in the ‘bypass’ and ‘secondary transfer’ groups was MVC, whilst falls <2m were most common in the ‘self-presented’ and ‘nearest’ groups. There was a significantly greater proportion of MVC patients in the ‘bypass’ group, compared to a higher proportion of low distance (<2m) falls and blows in the ‘secondary transfer’ group. The incidence of AIS ≥3 head injury was comparable between the ‘bypass’ and ‘secondary transfer’ groups; however, the ‘bypass’ group had a higher proportion of chest and limb injuries. The ‘self-presented’ and ‘nearest’ groups had a lower incidence of AIS ≥3 head injury and consisted of predominantly limb injuries. The ‘bypass’ group had a significantly greater mean number of body regions with an AIS ≥3 injury compared to other groups.

**Figure 2:**
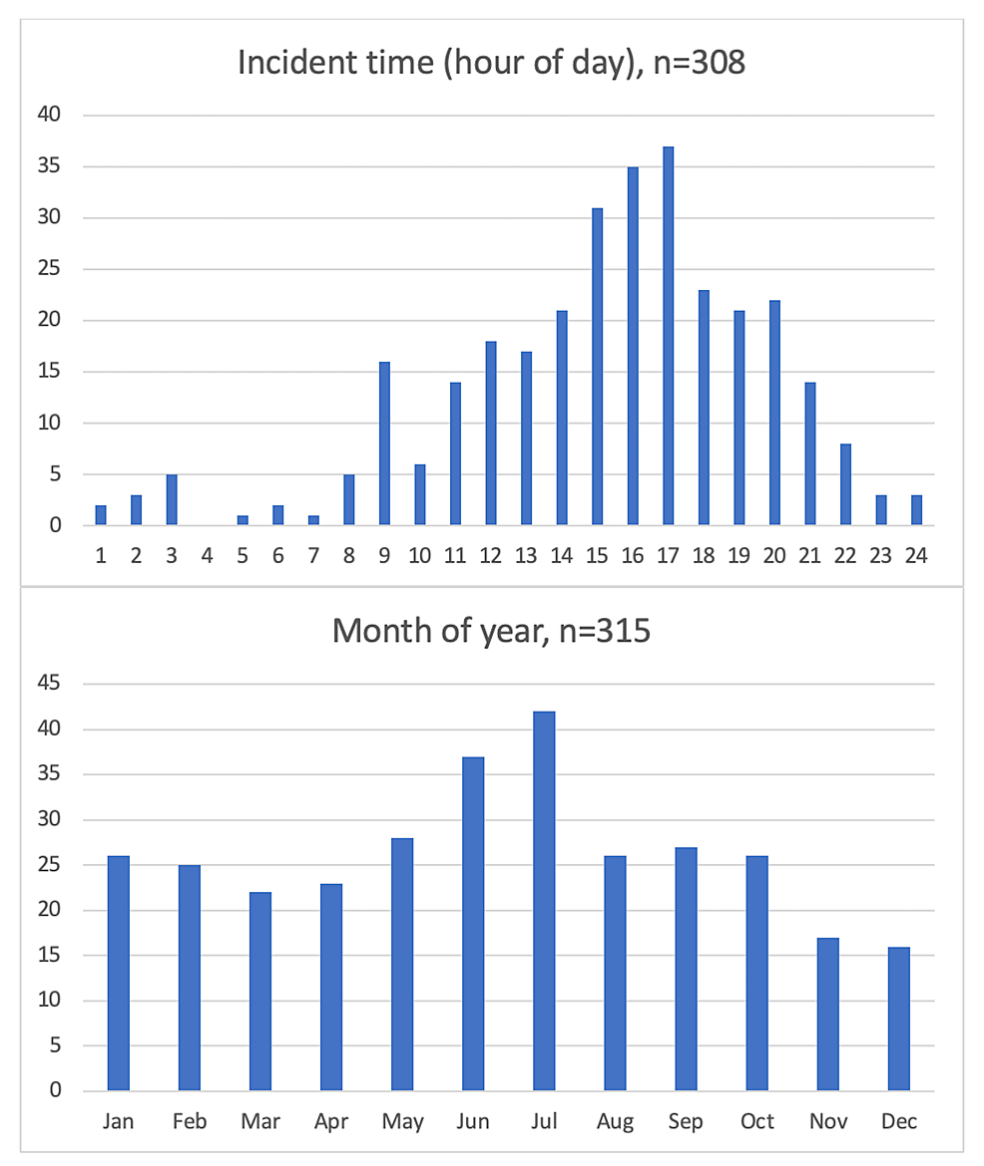
Incident time and month of presentation for paediatric trauma patients managed in the East of England Major Trauma Centre (MTC), 2015-2020

The highest incidence of paediatric trauma was observed in the late afternoon (1500-1700), and in the summer months (June, July) (Figure 2).

**Figure 3:**
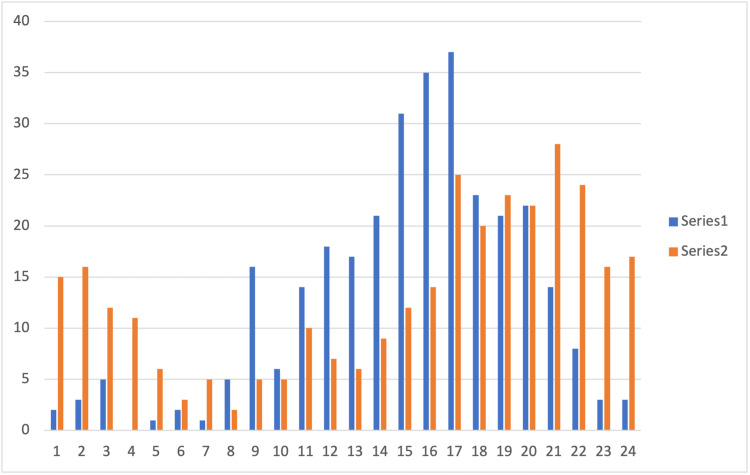
Incident and arrival time for paediatric trauma patients managed in the East of England Major Trauma Centre (MTC), 2015-2020 Series 1 = Incident time Series 2 = Arrival time

Whilst children are injured in the late afternoon they most often arrive at the MTC between 2100-0400 (Figure 3).

Mortality

The overall mortality was n=10/232 (4.3%). There was no significant difference in mortality between the two relevant groups: ‘bypass’ n=3/58 (5.2%), ‘secondary transfer’ n=7/174 (4.0%), OR 1.3 (95% CI 0.4-5.0), p=0.71.

Functional neurological outcome

The proportion of patients with a good functional neurological outcome (GOS=5) was significantly higher in the ‘secondary transfer’ group compared to the ‘bypass’ group (Table 2).

**Table 2:** A comparison of functional neurological outcome by mode of presentation for paediatric trauma patients managed in the East of England major trauma centre (2015-2020) GOS: Glasgow Outcome Scale

GOS	Self-presented	Nearest	Bypass	Secondary transfer
n	28	55	58	174
1 (death) / n (%)	0	6 (10.9%)	3 (5.2%)	7 (4.0%)
2 / n (%)	0	0	0	0
3 / n (%)	0	2 (3.6%)	4 (6.9%)	1 (0.6%)
4 / n (%)	0	11 (20.0%)	21 (36.2%)	28 (16.1%)
5 (good) / n (%)	28 (100.0%)	36 (65.5%)	31 (53.4%)	137 (78.7%)

Hospital and ICU length of stay

The ‘bypass’ group had a significantly longer hospital LOS compared to the ‘secondary transfer’ group - 8.5 [6.0-19.0] days and 5.0 [3.0-10.0] days, respectively, p<0.0001. A similar proportion of patients in both groups were admitted to ICU at the MTC: n=48/58 (82.8%) ‘bypass’, and n=126/174 (72.4%) ‘secondary transfer’ (OR 1.8 (95% CI 0.9-3.7), p=0.16). There was a statistically significantly longer ICU LOS in the ‘bypass’ group than the ‘secondary transfer’ group (2.0 [1.0-6.0] days and 1.0 [1.0-3.0] days respectively, p=0.0006).

Time to definitive care

Patients in the ‘bypass’ group had a significantly shorter time to definitive care compared to the ‘secondary transfer’ group - 117.6 [100.8-136.8] minutes and 418.8 [315.6-529.8] minutes, respectively, p<0.0001. On average, patients undergoing a ‘secondary transfer’ reached definitive care approximately five hours (301.2 minutes) later than patients in the ‘bypass’ group.

## Discussion

This study demonstrated no significant difference in mortality for paediatric major trauma patients who underwent ‘secondary transfer’ compared to those transported directly from the scene to the MTC, bypassing a nearer TU. The mortality in the overall cohort was low. However, patients in the ‘bypass’ group had a longer hospital and ICU LOS, and worse functional neurological outcome, despite reaching definitive care more than five hours sooner than patients undergoing ‘secondary transfer’. There was no difference in anatomical injury burden (ISS) between the ‘bypass’ and ‘secondary transfer’ groups. However, the ‘bypass’ group had a significantly greater proportion of polytrauma (greater mean number of AIS ≥3 body region injuries), chest and limb injuries, and higher energy mechanisms (MVC) compared to the ‘secondary transfer’ group. Whilst more generally, the most frequent MTC arrival time was observed to be between 21:00 - 04:00 is an important consideration for resource allocation.

Nearly half of all emergency presentations to UK paediatric emergency departments follow acute injury, most with minor injuries [5]. However, a small proportion of patients have serious traumatic injury. In this study, the most common presenting mechanism of injury was MVC, followed by fall <2m, with a low incidence of penetrating trauma, consistent with large national UK trauma registry data [5]. Mortality from paediatric trauma is low, and this study reports an overall mortality rate of 4.3%, with no significant difference in mortality between the two relevant groups (bypass n=3/58 (5.2%), secondary transfer n=7/174 (4.0%), OR 1.3 (95% CI 0.4-5.0), p=0.7)]. The observed mortality in this study is more than the national average of 3.1% [16,17]. However, the single-centre approach in this study is vulnerable to a selection bias of the most seriously injured children, demonstrated by the high proportion of major trauma patients (ISS >15), n=162/232 (69.8%). The lack of difference between the two transfer groups most likely reflects the overall appropriate use of the inclusive trauma network, similarities between cohorts, and the mechanism of injury confounding the decision to bypass or transfer.

Mortality in paediatric trauma has been explored by the Difference in access to Emergency Paediatric Intensive Care and care during Transport (DEPICT) study group, who also demonstrated no significant difference in 30-day mortality for patients transferred by a dedicated transfer team [8,9]. However, trauma was only a small subgroup of the DEPICT cohort, and crude mortality rates may not be the most illustrative outcome on the impact of paediatric transfer and retrieval given that death is a rare outcome, possibly representing type-2 error; functional outcomes may better represent the true impact.

There is a paucity of evidence exploring functional outcomes related to transfer status in paediatric trauma. This study has demonstrated a significantly worse functional neurological outcome in patients who ‘bypass’ the nearest hospital and are transported directly to the MTC in comparison to those undergoing ‘secondary transfer’. Whilst the number of patients with poor functional outcomes was low in the overall cohort and no significant difference in the proportion of patients with serious head injuries in each group, the ‘bypass’ cohort were more likely to have polytrauma with associated head and chest injuries. It is well understood that patients with the combination of brain and chest injuries have poorer outcomes than in isolation, which may explain the difference observed [5].

Data on ICU LOS and time to definitive care based on ‘transfer’ status [16,17] are predominantly from the United States [18-26], Canada [16,27,28], or Sweden [29], and report contrasting results which have limited generalisability to a UK trauma system due to differences in geography, trauma networks, and triage systems. Similar to the functional outcome analysis, longer hospital and ICU LOS in the ‘bypass’ groups are likely to be due to the differences in injury burden between the two groups in this study; however, these are comparable with the UK national average [5].

Some seriously injured children are not brought to the hospital by EMS, often presenting with caregivers at their nearest hospital. This study demonstrates that patients who undergo ‘secondary transfer’ arrive at definitive care more than five hours later than patients who are transported directly to the MTC. International studies report similar findings to this study’s paediatric trauma population, that although transferred patients had longer time to definitive care, there are no significant differences in the mortality, functional outcome, or complication rates between transferred and bypass patients, even after adjusting for injury severity [17,29]. However, a recent UK study including both adult and paediatric patients demonstrated that increased time to definitive care was associated with significantly delayed imaging, delayed surgery, and increased mortality [3]. The consistent message from these studies appears to demonstrate large differences in time to definitive care for patients requiring secondary transfer, and therefore prehospital trauma networks must ensure the early activation of transfer systems is in place to reduce any potential disadvantage in the paediatric population.

Limitations

Owing to data access constraints across multiple sites, these data were obtained from a single MTC in the East of England region, potentially resulting in a selection bias of the most seriously injured patients across a 20,000 km^2^ inclusive trauma network [12]. The study was also limited by the nature of a retrospective review with some missing data (including physiological data) and a chance of reporting bias. The TARN database only captures patients who are admitted to a hospital for more than 72 hours, admitted to an intensive care area, or who die in the hospital. Therefore, the impact of secondary transfers on patients outside of these groups is not known.

## Conclusions

This novel service evaluation provides a comprehensive overview of paediatric trauma outcomes related to transfer status. There was no significant difference in 30-day mortality of paediatric major trauma patients who underwent ‘secondary transfer’ compared to those transported directly from the scene to the MTC. However, patients in the ‘bypass’ group had a longer hospital and ICU LOS, and worse functional neurological outcome, despite reaching definitive care more than five hours sooner than patients undergoing ‘secondary transfer’, indicating the necessity of decisions to bypass. These results are useful in informing transfer decisions and encouraging ground ambulance crews to appropriately bypass.
